# Voltage sensors

**DOI:** 10.1016/j.molpha.2024.100011

**Published:** 2024-12-12

**Authors:** Lily Jan

**Affiliations:** Departments of Physiology, Biochemistry and Biophysics, Howard Hughes Medical Institute, University of California, San Francisco, California

**Keywords:** Voltage-gated sodium channel, Voltage-gated calcium channel, Voltage-gated potassium channel, Voltage-gated proton channel

## Abstract

Widely distributed in all kingdoms of life, voltage sensors in the membrane serve important functions via their movements driven by changes in voltage across the membrane (membrane potential). A voltage sensor domain contains 4 transmembrane segments (S1–S4). The S1–S3 helices form a hydrophobic constriction site (HCS, also known as the gating charge transfer center) that spans roughly one-third of the membrane thickness. Flanked by aqueous vestibules connected to the extracellular solution above the HCS or cytoplasmic solution below the HCS, the HCS forms a gating pore for the S4 segment bearing multiple basic residues. Membrane potential changes cause S4 to move through the HCS in a 3_10_ helical conformation. This S4 translocation generates a gating current as the positively charged S4 basic residues traverse the membrane electric field, transferring these gating charges from one aqueous vestibule to the other. For voltage-gated ion channels with their voltage sensor domains connected to pore domains, the HCS in the voltage sensor domain allows S4 but not ions to go through, while the channel pore formed by the pore domains mediates ion permeation. Voltage sensor mutations could result in *ω* currents that are conducted through the gating pore of mutant voltage-gated ion channels. These *ω* currents may cause pathological consequences in patients with periodic paralysis. Besides voltage-gated ion channels, the sperm-specific Na^+^/H^+^ exchanger and voltage-sensing phosphatases contain voltage sensors for membrane potential regulation. Notably, voltage-gated proton channels that are important for pH homeostasis are formed solely by the voltage sensor domain, which mediates proton permeation.

**Significance Statement:**

Voltage sensors mediate voltage regulation of ion channels, transporters, and phosphatases. The voltage sensor domain composed of 4 transmembrane segments (S1–S4) focuses the membrane electric field to the hydrophobic constriction site. To mediate voltage regulation, S4 basic residues within a 3_10_ helix move across the hydrophobic constriction site without concurrent ion flow through this gating pore. As a counterexample, voltage-gated proton channels are formed by the voltage sensor to mediate proton permeation. These ingeniously engineered voltage sensors are conserved throughout evolution.

## 1 Introduction

Hodgkin and Huxley made the prediction that membrane potential changes drive charge movements, which give rise to gating currents and initiate sodium current and potassium current for action potential generation (Hodgkin et al, 1952; Hodgkin and Huxley, 1952a,b,c). This prediction was borne out by gating current measurements, first for sodium channels in the squid axon (Armstrong and Bezanilla, 1973) and calcium channels for excitation-contraction coupling in skeletal muscle (Schneider and Chandler, 1973). By now, gating currents have been characterized for potassium channels in the squid axon (White and Bezanilla, 1985) as well as a number of heterologously expressed ion channels and other membrane proteins with voltage sensors (Bezanilla, 2018; Catacuzzeno et al, 2023).

**Fig. 1:**
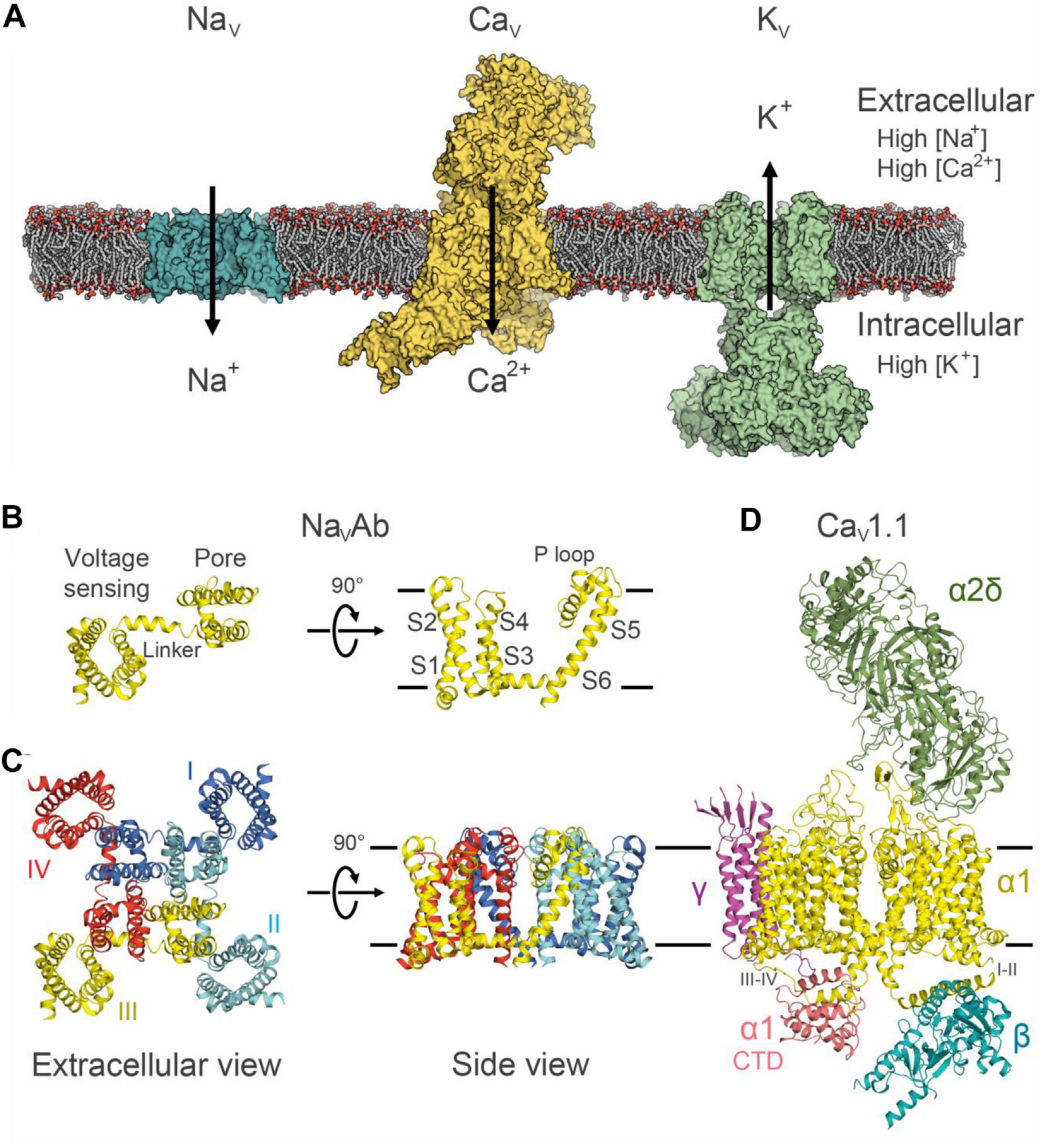
Voltage-gated sodium channels, calcium channels, and potassium channels. (A) Model of the bacterial sodium channel Na_V_Ab (cyan), the Ca_V_1.1 calcium channel complex (yellow), and the Kv1.2/2.1 chimeric potassium channel complex (green). (B) The structure of a subunit of the Na_V_Ab channel, with the voltage sensor domain (S1–S4) connected to the pore domain (S5–S6) via the S4–S5 linker. (C) The Na_V_Ab homotetrameric channel with domain swap. The pore is at the center of the channel, with the voltage sensor domain of one subunit interacting with the pore domain of a neighboring subunit. (D) Cryo-EM structure of the Ca_V_1.1 channel complex. This figure is from (Catterall et al, 2017) with permission of Nature Chemical Biology.

**Fig. 2:**
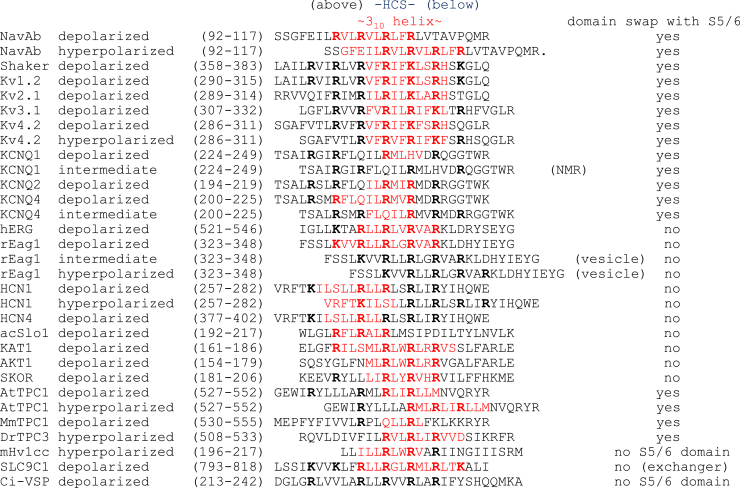
Voltage sensors with the approximated extent of 3_10_ helix (marked in red) and placement of S4 residues relative to the HCS in the middle of the membrane, based on structural analyses of membrane proteins, each with a single voltage sensor.

Catterall first achieved purification and reconstitution of voltage-gated sodium channels and calcium channels in the 1980s (Catterall, 1984; Takahashi et al, 1987; Catterall, 2023). The amino acid sequences of these channels provided the entryway to molecular cloning. Molecular studies of vertebrate voltage-gated sodium channels and calcium channels revealed that these channels are formed by a large *a* subunit with 4 homologous 6-transmembrane (TM) domains, each containing a voltage sensor domain with 4 TM segments (S1–S4) and a pore domain with 2 TM segments (S5-S6) (Noda et al, 1984; Tanabe et al, 1987; Catterall, 2010; Catterall et al, 2017) (Fig. 1). The S4 segment includes multiple basic residues that are at every third position, interspersed with 2 hydrophobic residues. This arrangement of the S4 residues inspired the sliding helix model (Catterall, 1986) and the helical screw model (Guy and Seetharamulu, 1986) for voltage sensor movements. Electrophysiological experiments combined with structural analyses have been carried out for decades to elucidate voltage sensor movements in a wide variety of voltage-gated ion channels and other membrane proteins with voltage sensors, such as the sperm-specific Na^+^/H^+^ exchanger (Windler et al, 2018; Kalienkova et al, 2023; Yeo et al, 2023) and voltage-sensing phosphatases (VSPs) that dephosphorylate phosphoinositide (PI) signaling lipids (Murata et al, 2005; Iwasaki et al, 2008; Halaszovich et al, 2009; Matsuda et al, 2011; Kurokawa et al, 2012; Sakata and Okamura, 2014) (Fig. 2).

**Fig. 3:**
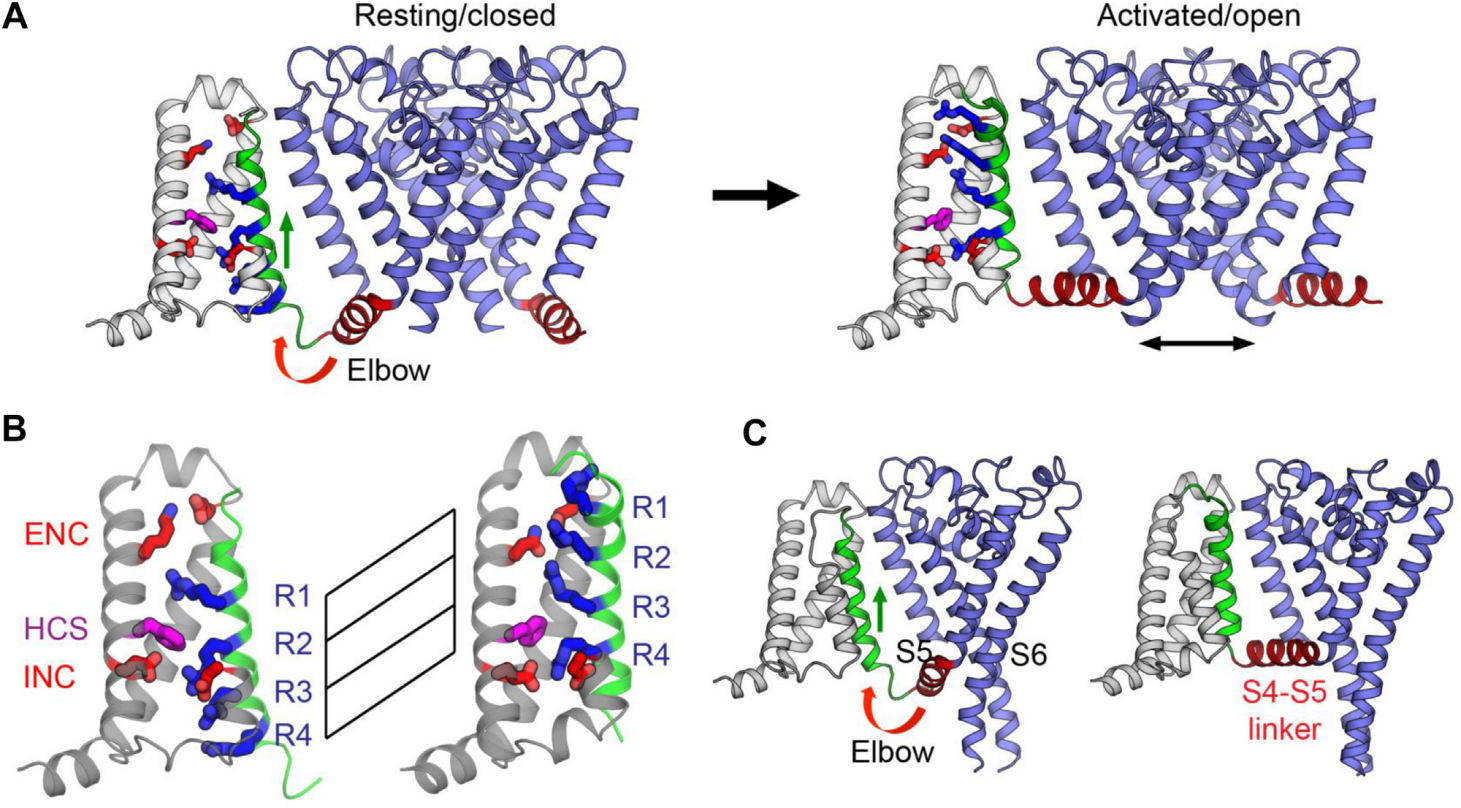
The bacterial sodium channel Na_V_Ab at the resting state and the activated state. (A) Structures in the resting/closed state (left) and activated/open state (right) are shown with only 1 voltage sensor for clarity, with S4 in green and its arginine residues (R1–R4) in blue. The E32 and N49 residues in the ENC and the acidic residues E59 and E80 in INC are in red, and the conserved phenylalanine at the HCS is in purple. The HCS in the voltage sensor domain forms a gating pore that allows S4 but not ions to go through, while the channel pore formed by the pore domains mediates ion permeation. Voltage sensor mutations could result in *ω* currents that are conducted through the gating pore of mutant voltage-gated ion channels (Gamal El-Din et al, 2014; Jiang et al, 2018). (B) Gating charge movement. S4 moves outward by 11.5 Å, passing 2 gating charges through the HCS. Part of S3 is omitted for clarity. (C) The outward movement of S4 (green) is accompanied by a slight rotation of S1–S3 segments and movement of the S4–S5 linker (red) for channel gating. This figure is from (Catterall et al. 2020) with permission of *Nature Chemical Biology*.

Structural analyses involving crystallography and/or cryogenic electron microscopy (cryo-EM) have provided snapshots of the locations of S4 basic residues with respect to the hydrophobic constriction site (HCS) that focuses the membrane electric field to about one-third of the membrane thickness. The S4 basic residues, which are more often arginine (R) than lysine (K), may interact with negatively charged aspartate (D) and/or glutamate (E) residues in the HCS that also contains a highly conserved S2 aromatic residue, which is more often phenylalanine (F) than tyrosine (Y). As shown in Figs. 2 and 3 for membrane proteins, each with a single S4 voltage sensor, the placement of S4 basic residues relative to the HCS varies with membrane potential, which is the voltage on the cytoplasmic side of the membrane relative to the conventional reference point in the extracellular solution.

Electrophysiological experiments combined with voltage clamp fluorometry and/or cysteine accessibility studies reveal multiple states for the voltage sensor, indicative of stepwise translocations of S4 basic residues. Voltage dependence of gating currents that reflect the movement of these S4 gating charges, as well as voltage dependence of the activity of voltage-gated ion channels or VSPs, may be altered via mutagenesis or signaling mechanisms. An increase in voltage sensitivity causes a left shift of the voltage dependence curve along the *x*–axis for membrane potential, whereas a right shift is indicative of a decrease in voltage sensitivity. Shifts of voltage dependence curves provide important clues in studies of voltage sensor function and regulation.

As a universal theme, depolarization (membrane potential changes that render the cytoplasmic side of the membrane more positive) drives S4 to move up toward the extracellular side of the membrane, while hyperpolarization (membrane potential changes that make the cytoplasmic side of the membrane more negative) causes S4 to move downward toward the cytoplasm. These S4 movements mediate voltage gating, albeit with different electrochemical coupling schemes for channels that open upon depolarization versus those that open upon hyperpolarization.

Starting with voltage-gated sodium channels, calcium channels, and potassium channels with similar structures (Catterall et al, 2017) (Fig. 1), this review will consider the diversity of electrochemical coupling in a variety of membrane proteins. Voltage sensors afford voltage regulation of not only ion channels that are activated by depolarization or hyperpolarization but also ion channels that are modulated by voltage and signaling moieties, ion channels that are formed by voltage sensors themselves, VSPs, as well as the sperm-specific Na^+^/H^+^ exchanger.

## 2 Voltage-gated sodium channels

Voltage-gated sodium channels initiate action potentials in neurons, muscles, and other excitable cells (Catterall, 2012). Most of the eukaryotic voltage-gated sodium channels are formed by a large *α* subunit containing 4 homologous 6-TM domains (*D*I–*D*IV), each with 6 TM segments (S1–S6) (Noda et al, 1984; Catterall, 2000). In contrast, bacterial sodium channels (Ren et al, 2001; Payandeh et al, 2011; Zhang et al, 2012; Payandeh and Minor, 2015) and the eukaryotic EukCatB sodium channel in haptophyte algae (Helliwell et al, 2020) are formed by 4 *α* subunits, each with 1 6-TM domain. The voltage sensor composed of S1–S4 of 1 subunit or homologous domain is adjacent to the pore domain containing S5 and S6 of an adjacent subunit or homologous domain, in a domain-swapped arrangement for voltage-gated sodium channels (Catterall, 2012) (Fig. 1).

The Na_v_1.5 voltage-gated sodium channel plays a critical role in the initiation of cardiac action potential in myocardial cells (Noble, 1984; Rogart et al, 1989; Fozzard and Hanck, 1996; Liu et al, 2003; DeMarco and Clancy, 2016). As the sole member on a branch of the sodium channel dendrogram (Goldin et al, 2000), cardiac Na_v_1.5 channels display structures (Jiang et al, 2020, 2021a) similar to those of neuronal and skeletal muscle sodium channels such as Na_v_1.2 (Pan et al, 2019), Na_v_1.4 (Pan et al, 2018), Na_v_1.6 (Li et al, 2023b), and Na_v_1.7 (Shen et al, 2019; Huang et al, 2022; Zhang et al, 2022), as well as bacterial sodium channels (Payandeh et al, 2011; Zhang et al, 2012).

Bacterial voltage-gated sodium channels present an excellent platform to elucidate the voltage sensor movements. The S4 basic residues that correspond to the gating charges move upward through the HCS upon depolarization in a stepwise manner to exchange their ion pair partners, which are acidic residues in the intracellular negative cluster (INC) or the extracellular negative cluster (ENC), as indicated by disulfide-locking experiments (DeCaen et al, 2008, 2009; Catterall, 2010; DeCaen et al, 2011). Structural studies reveal 2 states of the S4 voltage sensor in the Na_v_Ab bacterial sodium channel (Fig. 3). For Na_v_Ab bearing mutations that cause a left shift of the voltage dependence of channel activation as well as cysteine substitutions for disulfide bond formation, the S4 R2 (R102), R3 (R105), and R4 (R108) residues are below the HCS in the resting (hyperpolarized) state (Wisedchaisri et al, 2019). In contrast, the S4 R1 (R99), R2 (R102), and R3 (R105) residues are above the HCS in the activated (depolarized) state of Na_v_Ab channels (Payandeh et al, 2011; Catterall et al, 2017) (Fig. 2). In both resting and activated states, S4 includes a 3_10_ helical segment so that all 4 arginine residues are on the same side of S4 when they are in the vicinity of the HCS that includes the S2 F56 residue, the S1 I22 residue, and the S3 V84 residue (Payandeh et al, 2011; Wisedchaisri et al, 2019). The S4 segment moves by 2 helical turns upwards from the resting state to the activated state (Fig. 3). Electrophysiological and structural studies have delineated the cycle of conformation changes for voltage gating of Na_v_Ab channels (Catterall et al, 2020).

Gating charge movements in voltage-gated sodium channels with cysteine substitution of an S4 basic residue in 1 of the 4 homologous 6-TM domains affect the cysteine accessibility of the hydrophilic methanethiosulfonate reagents (Yang and Horn, 1995; Yang et al, 1996, 1997). These cysteine accessibility experiments reveal that the first 3 S4 gating charges (R1–R3) of *D*I and *D*II are accessible to the extracellular solution above the HCS in the activated state of Na_v_1.5 sodium channels. In the ENC above the HCS, acidic residues interact with the S4 R2 and R3 residues in *D*I or the S4 R1 and R2 residues in *D*II. In the INC below the HCS of *D*I and *D*II, acidic residues interact with the S4 K4 residue (Jiang et al, 2020). For *D*III and *D*IV, each with more than 4 S4 gating charges, R1–R4 are accessible to the extracellular solution above the HCS in the activated state of Na_v_1.5 voltage-gated sodium channels (Jiang et al, 2020). Structural analyses reveal a 3_10_ helix that includes the S4 gating charges R2 to R6 of *D*IV in the activated state of Na_v_1.5 sodium channels (Jiang et al, 2021b). Multiple studies suggest that S4 in *D*IV is involved in fast inactivation rather than activation of voltage-gated sodium channels, as summarized below.

First, binding of the deathstalker scorpion *α*-toxin LqhIII to the top of the S4 voltage sensor of *D*IV allows the K64 residue of this *α*-toxin to interact with the acidic residue that normally interacts with the S4 R1 and R2 residues in the ENC of *D*IV in the activated state of Na_v_1.5 sodium channels. Consequently, S4 of *D*IV in the toxin-bound Na_v_1.5 sodium channel shifts 2 helical turns downward from its normal post in the activated state. In this toxin-stabilized conformation, S4 in *D*IV forms a 3_10_ helix for the segment that includes the first 4 gating charges (R1–R4), with R1 and R2 interacting with 2 acidic residues in the ENC and R3 and R4 forming ion pairs with 2 acidic residues in the INC. This conformation with 4 ion pairs aligned on one side of S4 in *D*IV is compatible with voltage sensor movements in *D*I, *D*II, and *D*III to trigger channel opening while preventing fast inactivation of the toxin-bound Na_v_1.5 sodium channel (Jiang et al, 2021b). These findings are consistent with the scenario that the voltage sensor of *D*IV is involved in fast inactivation of cardiac voltage-gated sodium channels.

Second, studies of the Na_v_1.4 skeletal muscle voltage-gated sodium channel, which are based on single-channel analyses combined with voltage clamp fluorimetry for monitoring the movement of individual voltage sensors, indicate that activation of voltage sensors in *D*I–III causes the channel pore to fully open, whereas movement of the voltage sensor in *D*IV promotes pore rearrangements to a conformation that facilitates fast inactivation (Goldschen-Ohm et al, 2013). Moreover, negative charges on S1 or S2 in the ENC of *D*I and *D*II, but not *D*III or *D*IV, appear to destabilize the open conformation of Na_v_1.4 sodium channels (Pless et al, 2014). These studies support the notion that voltage sensor movements in the first 3 homologous domains mediate sodium channel activation upon depolarization while the voltage sensor movement in *D*IV is primarily involved in facilitating channel inactivation for skeletal muscle as well as cardiac voltage-gated sodium channels.

## 3 The *ω* current through the gating pore of voltage-gated ion channels with voltage sensor mutations

In voltage-gated sodium channels and calcium channels, gating charge translocation owing to S4 movements through the gating pore formed by the HCS is normally not accompanied by ion flow through these gating pores (Catterall, 2000, 2010). However, the gating pore in mutant Na_v_1.2 voltage-gated sodium channels with substitutions of a *D*II S4 arginine mediates ion flow, also known as the *ω* current (Sokolov et al, 2005). Notably, *ω* currents caused by S4 arginine substitutions in voltage-gated sodium channels and calcium channels could result in pathological consequences.

Hypokalemic periodic paralysis is associated with mutations of S4 arginine residues of the Na_v_1.4 sodium channel and the Ca_v_1.1 calcium channel in skeletal muscle (Venance et al, 2006). Gating pore-mediated *ω* currents are associated with the majority of such mutations of Ca_v_1.1 that cause hypokalemic periodic paralysis (Wu et al, 2021). In addition, mutations of the S4 R1 or R2 residue in *D*II of Na_v_1.4 cause hypokalemic periodic paralysis and induce hyperpolarization-activated cation current. The gating pore that conducts this leak *ω* current can be blocked by guanidinium ions that resemble the arginine sidechain, while depolarizations that cause voltage sensor activation eliminate this *ω* current in mutant Na_v_1.4 sodium channels (Sokolov et al, 2007, 2010). On the other hand, the hypokalemic periodic paralysis mutation R669H of the S4 R1 residue in *D*II of Na_v_1.4 causes the gating pore to allow proton permeation (Struyk and Cannon, 2007; Wu et al, 2011).

Notably, a wide spectrum of periodic paralysis is associated with mutations that cause *ω* current conduction via the gating pore in the voltage sensor domain. Normokalemic periodic paralysis is associated with mutations of the S4 R3 residue in *D*II of Na_v_1.4. These mutations result in *ω* current through the gating pore during depolarization for voltage sensor activation, in a conformation of the voltage sensor that is retained in the slow-inactivated state of the channel, while hyperpolarization that causes channel deactivation eliminates this *ω* current (Sokolov et al, 2008). Taken together, these findings indicate that replacement of 1 S4 arginine by amino acids with a smaller sidechain enables ion permeation through the gating pore under conditions that would normally place that S4 arginine in the gating pore.

For studies of periodic paralysis mutations in bacterial sodium channels, the S4 R2 or R3 residue of Na_v_Ab is replaced with glycine or cysteine, resulting in *ω* currents via the gating pore (Gamal El-Din et al, 2014; Jiang et al, 2018). Structural analyses reveal an aqueous cleft through the length of the activated voltage sensor domain of Na_v_Ab channels bearing the R3G mutation. Moreover, there is a binding pocket for guanidinium in the closed gating pore of the activated voltage sensor of Na_v_Ab channels bearing the R2G mutation (Jiang et al, 2018). These findings support the notion that S4 arginine residues prevent ion permeation via the gating pore during their translocation to control the pore opening of voltage-gated ion channels.

## 4 Voltage-gated calcium channels

Voltage-gated calcium channels regulate a variety of cellular processes, including contraction, secretion, neurotransmission, and gene expression. The Ca_v_1 family with 4 members represents L-type calcium channels that require strong depolarization for activation. The 3 Ca_v_2 family members correspond to the P/Q-type, N-type, and R-type calcium channels that also require strong depolarization for activation. These different types of high-voltage-activated calcium channels are pharmacologically distinguishable. As to the 3 Ca_v_3 family members, they are low-voltage-activated T-type calcium channels that conduct transient currents (Ertel et al, 2000).

The Ca_v_1.1 L-type voltage-gated calcium channels mediate excitation-contraction coupling in skeletal muscle. Four Ca_v_1.1 channels cluster at the triad junctions that are formed by the plasma membrane of the T-tubules transverse-tubule network and the sarcoplasmic reticulum (SR). These Ca_v_1.1 channels on the plasma membrane interact with ryanodine receptors in the SR to induce calcium release from the SR (Bibollet et al, 2023). Cryo-EM studies reveal that all S4 gating charges are on one side of a 3_10_ helix in each of the 4 voltage sensors of rabbit Ca_v_1.1 channels (Wu et al, 2016). Whereas S4 in *D*IV contains 4 gating charges (R1, R2, R3, K4), S4 in *D*I and *D*III includes an additional gating charge (K0) preceding R1–R4, while S4 in *D*II includes one (K5) following K4. The S4 (K0), R1, R2, and R3 residues are above the HCS, while the other gating charges are below the HCS of the Ca_v_1.1 channel in the presumed depolarized state (Wu et al, 2016; Fernández-Quintero et al, 2021).

Electrical interactions between S4 basic residues and acidic residues in the aqueous vestibules stabilize the voltage sensor. The S4 basic residues in *D*I and *D*III form ion pairs with 2 glutamate residues on S2, whereas the S4 basic residues in *D*II and *D*IV form ion pairs with 1 acidic residue on S2 and another on S3 of rabbit Ca_v_1.1 channels (Wu et al, 2016; Fernández-Quintero et al, 2021). Neutralization of the first S2 acidic residue (E87A) in *D*I of Ca_v_1.1 channels, which are expressed in transfected myotubes formed by the dysgenic (mdg/mdg) cell line GLT, reduces the voltage sensitivity by causing a right shift of the voltage dependence of activation without altering activation kinetics. Neutralization of the second S2 acidic residue (E90A) in *D*I causes acceleration of activation kinetics as well as the right shift of the voltage dependence curve (Fernández-Quintero et al, 2021). These findings implicate the S2 acidic residues in *D*I for stabilizing the voltage sensor. These S2 acidic residues may sequentially interact with different S4 basic residues during voltage-driven movement of the S4 voltage sensor.

Developmental regulation of excitation-contraction coupling likely arises from differences in the electrostatic interactions that stabilize the S4 gating charges. Unlike the adult splice isoform Ca_v_1.1a that includes exon 29 with 19 amino acids in the S3-S4 loop of *D*IV, the embryonic splice isoform Ca_v_1.1e lacks exon 29 and mediates faster SR release that is also more sensitive to depolarization (Tuluc et al, 2009). Ca_v_1.1e, but not Ca_v_1.1a, can sustain the electrostatic interactions of the S4 R1 and R2 residues with the S3 acidic residue (D1196) in the voltage sensor domain of *D*IV as well as the S5 acidic residue (E216) in the pore domain of *D*I. Owing to the domain swap arrangement of voltage-gated calcium channels (Fig. 1), the voltage sensor of *D*IV is adjacent to the pore domain of *D*I, thereby allowing S5 in the pore domain of *D*I as well as S3 in the voltage sensor domain of *D*IV to provide countercharges to the S4 gating charges in *D*IV (El Ghaleb et al, 2021).

The 4 voltage sensors of the Ca_v_1.1 channel have different functional roles in mediating excitation-contraction coupling that depends on physical interactions between Ca_v_1.1 channels and ryanodine receptors, involving the intracellular loop between *D*II and *D*III of Ca_v_1.1 channels (Tanabe et al, 1990; Nakai et al, 1998; Bannister and Beam, 2013). Action potential fluorometry, which is capable of tracking individual voltage sensor movements in skeletal muscle, detects movements of the S4 voltage sensors in *D*I, *D*II, and *D*IV, but not *D*III, of Ca_v_1.1 channels during an action potential and SR release of calcium (Banks et al, 2021).

The roles played by the 4 voltage sensors in causing calcium ion permeation through Ca_v_1.1 channels differ from their involvement in excitation-contraction coupling. As indicated by voltage clamp fluorometry combined with current recording from human Ca_v_1.1 channels that are expressed in *Xenopus* oocytes, activation of the S4 voltage sensor in *D*I is critical and likely rate-limiting for opening the Ca_v_1.1 calcium channel pore (Savalli et al, 2021). Consistent with the differential involvements of voltage sensors in controlling channel opening versus excitation-contraction coupling, opening of the Ca_v_1.1 calcium channel pore takes longer and requires stronger depolarization than the SR calcium release induced by voltage sensor movements of Ca_v_1.1 channels (Garcia et al, 1994).

Whereas Ca_v_1.1 channels mediate excitation-contraction coupling via their physical contact with ryanodine receptors rather than calcium permeation, other Ca_v_ family members function primarily as voltage-gated calcium channels. As indicated by voltage clamp fluorometry combined with current recordings from human Ca_v_1.2 channels expressed in *Xenopus* oocytes, movements of S4 voltage sensors in *D*II and *D*III play a major role and are rate-limiting for channel activation (Pantazis et al, 2014). Neutralization of some or all of the S4 basic residues in individual homologous 6-TM domains implicates S4 in *D*I and *D*III to a greater extent than S4 in *D*II for voltage gating of Ca_v_1.2 channel opening, while the S4 voltage sensors in *D*I, *D*II, and *D*III all modulate the stability of channel gate opening and closing (Beyl et al, 2016). These studies support the possibility of asynchronous movements of voltage sensors so that a subset of these movements would be rate-limiting.

Cryo-EM studies reveal similar arrangements of S4 voltage sensors for Ca_v_1.1, Ca_v_1.3, and Ca_v_3 family members, with all 4 voltage sensors in the up or activated state (Wu et al, 2016; Zhao et al, 2019; Yao et al, 2022a, 2024). In contrast, structural analyses of Ca_v_1.2, Ca_v_2.2, and Ca_v_2.3 channels find the voltage sensor of *D*II in the down or resting state (Gao et al, 2021; Yao et al, 2022b; Gao et al, 2023a; Yao et al, 2024).

For the N-type Ca_v_2.2 channel, the entire S4 TM segment of *D*II is in a 3_10_ helix and is shifted downward from the activated state, so that 1 S4 basic residue (R2) is above the HCS and the other 4 S4 basic residues (R3–K6) are below the HCS. As to the other 3 voltage sensors of Ca_v_2.2, the first 4 S4 basic residues in *D*I (R1, R2, R3, and R4) and *D*III (K1, R2, R3, and R4) and the first 3 S4 basic residues in *D*IV (R2, R3, and R5) are above the HCS, while the other S4 basic residues (K5 in *D*I, K5 and K6 in *D*III, and K5 and R6 in *D*IV) are below the HCS. The top of S4 in these homologous 6-TM domains is composed of 1 or 2 turns of *α* helix, while the rest of S4 forms a 3_10_ helix (Dong et al, 2021; Gao et al, 2021; Yao et al, 2024). The 3_10_ helical arrangement allows positively charged residues to be aligned on one side as S4 moves through the HCS.

For the R-type Ca_v_2.3 channel, the S4 segments also form 3_10_ helices harboring the gating charges. While the S4 voltage sensor in *D*II is in the down or resting state, the other 3 voltage sensors are in the up or activated state (Gao et al, 2023b). Neutralization of S4 basic residues in *D*I, *D*III, or *D*IV abolishes Ca_v_2.3 channel activation. Glutamine substitutions of the 3 arginine residues and 1 lysine on S4 in *D*II cause a left shift of the voltage dependence of activation as well as steady-state inactivation, indicating that the S4 voltage sensor in *D*II of Ca_v_2.3 calcium channels may be involved in voltage modulation of channel properties rather than voltage gating for channel activation (Gao et al, 2023b).

Differences in the voltage threshold for channel activation could conceivably arise from differences in voltage sensors and/or other aspects of electrochemical coupling. For example, the low-voltage-activated T-type Ca_v_3.3 calcium channel has its pore-lining S6 helix of *D*III extended into the cytoplasm to include a positively charged segment with 6 helical turns. This extended S6 helix of *D*III directly interacts with the S4 voltage sensor of *D*IV (He et al, 2022). Neutralization of all 12 basic residues in this cytoplasmic extension of the S6 helix of *D*III or glycine substitutions of 3 residues preceding this cytoplasmic extension causes right shifts of the voltage dependence of activation. It thus appears that the positively charged residues in this extended S6 helix of *D*III contribute to the greater voltage sensitivity of Ca_v_3.3 channels, even though the number of gating charges in each S4 voltage sensor of the low-voltage-activated Ca_v_3.3 calcium channel is identical to that of the high-voltage-activated Ca_v_2.2 calcium channel (He et al, 2022).

## 5 Voltage-gated potassium channels in the Kv1–Kv4 families

The core voltage-gated potassium channel families for neuronal signaling include low-voltage activated potassium channels in the Kv1 and Kv4 families and high-voltage activated potassium channels in the Kv2 and Kv3 families. These potassium channels control the threshold and waveform of action potentials in neurons (Johnston et al, 2010).

Voltage gating of Kv1 channels has been extensively studied. The first 4 S4 arginine residues (R1–R4) contribute to the gating charge movements during the activation of Shaker channels (Aggarwal and MacKinnon, 1996; Seoh et al, 1996) and Kv1.2 channels (Ishida et al, 2015). Membrane potential changes alter the accessibility of sulfhydryl-modifying agents in the extracellular solution to cysteines that replace various S4 residues, revealing that depolarization causes S4 to move upward and expose those S4 residues to the extracellular solution (Larsson et al, 1996; Yusaf et al, 1996; Baker et al, 1998). Moreover, histidine scanning of S4 arginine residues indicates that the S4 R1 residue resides at the HCS, also known as the gating pore or the gating charge transfer center (Islas and Sigworth, 2001; Tao et al, 2010), in the resting (hyperpolarized) state. In contrast, the S4 R4 residue may reside at the HCS in the activated (depolarized) state of the Shaker potassium channel (Starace et al, 1997; Starace and Bezanilla, 2001, 2004).

Structural analyses have characterized Kv1 channels at depolarized membrane potential (Fig. 2). The HCS in the voltage sensor domains of Kv1.2 channels corresponds to a hydrophobic layer of about 10 Å in length, at the midpoint of the membrane bilayer. It includes 10 completely buried residues that are predominantly hydrophobic: V172, I173, S176, I177, and F180 on S1; C229, I230, and F233 on S2; along with A262 and I263 on S3 (Chen et al, 2010). At the presumed activated (depolarized) state of Kv1.2 potassium channels, the S4 R4 and K5 residues localize to the HCS as do the highly conserved acidic residues E226 on S2 and D259 on S3 (Long et al, 2005, 2007; Chen et al, 2010; Y. Wu, preprint, DOI: https://doi.org/10.1101/2023.06.02.543446), similar to the voltage sensor organization of Shaker potassium channels (Tan et al, 2022). Electrostatic interactions of the S4 R3 and R4 residues with a highly conserved S2 acidic residue nearby stabilize the open state, as indicated by mutagenesis studies (Long et al, 2007; Pless et al, 2011; Y. Wu, preprint, DOI: https://doi.org/10.1101/2023.06.02.543446).

A recurrent theme for voltage sensors in voltage-gated ion channels is the presence of a 3_10_ helix. The S4 R4, K5, and R6 residues are within a stretch of 3_10_ helix in Kv1.2 channels in the active (depolarized) state (Long et al, 2005, 2007; Chen et al, 2010), aligning these gating charges on one side for S4 translocation through the HCS. In a test for the functional significance of the S4 secondary structure rearrangements, individual S4 residues are substituted with *α*-hydroxy acid that replaces the backbone amide with an ester, thereby eliminating the capacity for hydrogen bond formation. Voltage gating of Kv1.2 channels is impaired by substitutions of S4 residues with this unnatural amino acid at the transition point of S4 between the *α*-helical segment and the 3_10_-helical segment in the active state (Infield et al, 2018). These findings support the notion that voltage sensor movements involve rearrangements of S4 from the 3_10_ helix for translocation through the HCS to the less energetically costly *α* helix for the S4 segment that is far from the HCS.

Gating current measurements of Shaker potassium channels expressed in *Xenopus* oocytes reveal gating charge movements among closed states (Bezanilla, 2018). The voltage sensor likely takes on various intermediate positions in multiple closed states (Zagotta et al, 1994; Baker et al, 1998; Schoppa and Sigworth, 1998). Cd^2+^ bridging of S3 and S4 with cysteine substitutions reveals extensive movement of S4 relative to S3. Whereas the S4 basic residues R1–R4 are above the HCS in the open (depolarized) state, stepwise downward movements of S4 would bring all 4 S4 arginine residues below the HCS in the deep closed (hyperpolarized) state of Shaker channels (Henrion et al, 2012).

Consistent with the notion that the membrane electric field drives movements of S4 gating charges through the narrow HCS, substitution of the S4 R1 residue of Shaker channels with smaller amino acids results in *ω* currents that are prominent when the voltage sensor is at the resting state at hyperpolarized membrane potentials. This *ω* current can be carried by cations, including guanidinium ions that resemble the arginine sidechain (Tombola et al, 2005). Remarkably, a truncated Shaker protein that retains the voltage sensor domain but not the pore domain gives rise to *ω* currents at hyperpolarized membrane potentials, indicating that the pore domain exerts an allosteric influence to prevent the voltage sensor domain from generating *ω* currents for proton permeation (Zhao and Blunck, 2016).

As in the case of Kv1.2 channels, Kv2.1 channels have an HCS that includes the highly conserved phenylalanine on S2 and 2 highly conserved acidic residues on S2 and S3. The S4 R1–R3 residues are above the HCS, while the S4 K4 and R5 residues are near the HCS in this depolarized state of Kv2.1 channels (Fernández-Mariño et al, 2023) (Fig. 2). Cysteine substitutions and Cd^2+^ bridging experiments support the notion that the S4 basic residues, including R1, move upwards through the HCS upon Kv2.1 channel activation (Fernández-Mariño et al, 2023).

The Kv3.1 potassium channels have the S4 R1–R3 residues above the HCS, while the S4 R4 residue displays cation-*π* interactions with the highly conserved S2 phenylalanine in the HCS (Botte et al, 2022; Chi et al, 2022) (Fig. 2). Interaction between the backbone carbonyl of the S4 R6 residue with arginine in the S4–S5 linker, as well as interaction between the C-terminal helix of the cytoplasmic T1 domain and lysine in the S6 extension, likely contribute to the electrochemical coupling for voltage sensor movements to trigger channel activation. These extensive interactions may partially account for the tighter electromechanical coupling of the high-voltage-activated Kv3 channels (Chi et al, 2022).

The Kv4.2 potassium channels in the open conformation have the S4 R1–R3 residues above the HCS, while K4 is within the HCS (Kise et al, 2021; Ye et al, 2022). In the closed conformation stabilized by Hg^2+^ bridging of cysteine substitutions near the C-terminal end of the pore-lining S6 helices, the S4 R3 residue resides in the HCS while K4 is below the HCS, and the S4–S5 linker is displaced by 7 Å toward the intracellular side (Ye et al, 2022). The 3_10_-helical region of S4 spans a similar range in the membrane for Kv4.2 channels in the open or closed conformation (Fig. 2), indicating that S4 movements involve reorganization of its secondary structure so that residues near the top of the 3_10_-helical segment take on the *α* helical conformation as S4 moves up toward the extracellular side of the membrane (Ye et al, 2022)—a concertina-like movement (Long et al, 2007).

Voltage-gated potassium channels that control neuronal excitability share common features for voltage gating (Johnston et al, 2010; Jan and Jan, 2012; Ovsepian et al, 2016; Villa and Combi, 2016). For example, the interaction between a conserved S1 acidic residue (E226) and the S4 R4 (R303) residue of Kv1.2 channels is similar to the interaction between the equivalent S1 acidic residue (E249) and the S4 R3 (R317) residue of Kv3.1 channels (Chi et al, 2022). As indicated by structural analyses of these voltage-gated potassium channels in the open conformation, voltage sensor activation via depolarization might involve the translocation of 4 S4 arginine residues of Kv1 channels, or 3 S4 arginine residues of Kv2, Kv3, or Kv4 channels, to the extracellular side of HCS, also known as the gating charge transfer center or the gating pore (Islas and Sigworth, 2001; Tao et al, 2010) (Fig. 2).

## 6 The KCNQ (Kv7) family of voltage-gated potassium channels

Action potential repolarization in the mammalian myocardium involves the transient outward K^+^ current (I_to_) mediated by Kv4 channels as well as delayed rectifier K^+^ currents with 2 prominent components, I_Kr_ (I_K, rapid_) and I_Ks_ (I_K, slow_) (Nerbonne and Kass, 2005). The cardiac I_Ks_ current is mediated by KCNQ1 (Kv7.1) in complex with KCNE1 with potentially variable stoichiometry (Nerbonne and Kass, 2005; Dixit et al, 2020; Wang et al, 2020).

At the resting (hyperpolarized) state of KCNQ1 potassium channels, the S1 E160 residue interacts with the S4 R1 (R228) residue (Wu et al, 2010). Depolarization drives stepwise upward movements of the S4 voltage sensor, first to the intermediate state and then to the activated state. The charge-reversing mutations E160R on S1 combined with R231E on S4 (E1R/R2E) lock the voltage sensor in the intermediate state, while the charge-reversal mutations E160R along with R237E (E1R/R4E) lock the voltage sensor in the activated state (Wu et al, 2010; Barro-Soria et al, 2014; Nakajo and Kubo, 2014; Zaydman et al, 2014). These findings indicate that the S1 E160 residue switches partners for electrostatic interactions as the S4 gating charges move upwards.

Structural analyses provide further evidence for stepwise S4 movements. For the KCNQ1 voltage sensor in the intermediate state, NMR analyses reveal pairing of the S4 R2 (R231) residue with the S1 E160 residue as well as interactions of the S4 R4 (R237) residue with the S2 E170 residue and the S3 D202 residue in the HCS, while the S4 Q234 residue interacts with the S2 F167 residue that acts as the aromatic plug of the HCS (Taylor et al, 2020). As to the activated state of the KCNQ1 voltage sensor, cryo-EM studies reveal interaction between the S4 R4 (R237) residue and the S1 E160 residue above the HCS, while the S4 H240 residue resides in the HCS and the S4 R6 (R243) residue is just below the HCS (Sun and MacKinnon, 2017, 2020; Ma et al, 2022; Mandala and MacKinnon, 2023) (Fig. 2). Cryo-EM studies of KCNQ1 in liposomes further suggest that the placement of the voltage sensor at the resting state would have prevented phosphatidylinositol 4,5-bisphosphate (PIP_2_) association for channel activation (Mandala and MacKinnon, 2023).

The KCNQ1 channel may open when a single voltage sensor reaches the intermediate state, albeit with a greater energy barrier to overcome (Westhoff et al, 2019; Wang et al, 2020). Whereas KCNQ1 channel opening with its voltage sensor at the intermediate state is triggered by interaction between the S4–S5 linker and the pore domain of the same subunit, KCNQ1 channel opening with its voltage sensor at the activated state involves interactions of S4 residues above the HCS as well as the N-terminal S4–S5 linker with the pore domain of a neighboring subunit, which is physically adjacent to this voltage sensor (Hou et al, 2020). Thus, the multiple steps of voltage sensor translocations may trigger voltage sensor interactions with the pore domain of the same subunit or a neighboring subunit to cause channel opening in multiple stages.

Structural analyses of other KCNQ family members have captured S4 at various locations relative to the HCS (Fig. 2). The KCNQ2 voltage sensor in the activated state has the S4 R198, R201, and R207 residues above the HCS, while the S4 R210 residue is within the HCS that includes the S2 F137 and E140 residues and the S3 D172 residue (Li et al, 2021b). For the open conformation of the KCNQ4 channel in complex with the channel modulator ML213 and PIP_2_, the voltage sensor is in the activated state, with the S4 R1, R2, Q3, and R4 residues above the HCS (Zheng et al, 2022) (Fig. 2). In contrast, the voltage sensor of KCNQ4 channels in the closed conformation is in an intermediate state. In this case, the S4 R1, R2, and Q3 residues are above the HCS, R4 is at the HCS, while R5 and R6 are below the HCS (Li et al, 2021a)—1 3_10_-helical turn downward from the S4 placement in the activated state (Fig. 2).

## 7 Variations of electrochemical coupling between the voltage sensor and the pore domain

Voltage gating may involve electrochemical coupling in multiple ways. The Shaker-like voltage-gated potassium channels mainly rely on interactions between the S4–S5 linker and the cytoplasmic end of S6 for voltage sensor movements to control pore opening (Blunck and Batulan, 2012). However, electrochemical coupling for Shaker potassium channels also involves interactions between the voltage sensor of 1 subunit and its adjacent pore domain from a neighboring subunit, at locations near the extracellular side of the membrane (Bassetto et al, 2021). It seems likely that channel gating involves multiple ways of electrochemical coupling.

KCNQ channels exemplify the myriad ways of electrochemical coupling (Hou et al, 2020; Wang et al, 2020; Ma et al, 2022). The canonical electrochemical coupling that involves interactions between the S4–S5 linker and the cytoplasmic end of S6 promotes pore opening with the voltage sensor in the intermediate state of activation. KCNE1 interaction with KCNQ1 prevents pore opening at this intermediate state but is permissible for pore opening in the activated state that involves interactions of S4 as well as the N-terminal S4–S5 linker of 1 subunit with the pore-lining S6 helix from a neighboring subunit (Hou et al, 2020). Moreover, KCNQ1 channel pore opening requires the presence of PIP_2_, which binds at the cleft between the voltage sensor and the pore domain (Ma et al, 2022).

Channel gating could be further modified by auxiliary subunits and modulators. For example, binding of the KCNQ1 channel activator ML277 induces an upward movement of the S4–S5 linker and pore opening, even in the absence of PIP_2_ (Xu et al, 2015; Ma et al, 2022). Whereas KCNE1 association modifies KCNQ1 channel kinetics, KCNQ1 channels assembled with the KCNE2 or KCNE3 auxiliary subunits are constitutively open, a feature of potential physiological importance to epithelial ion homeostasis (Wang et al, 2020).

The Kv1–Kv7 families of voltage-gated potassium channels display domain swap between the voltage sensor domain and the pore domain in a way analogous to voltage-gated sodium channels and calcium channels (Fig. 1). Whereas the Kv1-Kv9 families of potassium channels have a helical S4–S5 linker of ∼15 amino acids that connects the voltage sensor with the pore domain, voltage-gated potassium channels in the Kv10–Kv12 families have a much shorter S4–S5 linker of ∼6 amino acids (Whicher and MacKinnon, 2016, 2019). There is no domain swap between the voltage sensor domain and pore domain in the Kv10-Kv12 families of voltage-gated potassium channels as well as the hyperpolarization-activated cyclic nucleotide–gated (HCN) family of HCN cation channels. With the voltage sensor adjacent to the pore domain of the same subunit, these channels tend to display variations in electrochemical coupling, while their pore-forming *α* subunits may be amenable to modulation by calcium ions or signaling molecules.

## 8 The hERG (Kv11.1) voltage-gated potassium channel

The I_Kr_ current for cardiac action potential repolarization is mediated by hERG channels (encoded by the human Ether-à-go-go-related gene) (Nerbonne and Kass, 2005). The hERG channels display slow activation and deactivation but fast inactivation. These kinetic properties allow hERG channels to recover from inactivation during the falling phase of cardiac action potential to regulate repolarization (Zequn and Jiangfang, 2021; Sanchez-Conde et al, 2022).

Cryo-EM analyses of the hERG channel reveal an open pore surrounded by S6 helices bearing a glycine hinge (G648) (Wang and MacKinnon, 2017). A very short S4–S5 linker connects the voltage sensor domain with the pore domain without displaying domain swap. The first 3 S4 basic residues (K525, R528, and R531) contribute most of the gating charges (Zhang et al, 2004) and are above the HCS of the hERG channel in the activated (depolarized) state, while the S4 R4 (R534) and R5 (R537) residues are below the HCS (Wang and MacKinnon, 2017) (Fig. 2).

Given the voltage-dependent placement of S4 relative to the HCS, mutations of an S4 basic residue may cause *ω* currents with a voltage dependence determined by the location of that S4 basic residue with respect to the HCS that focuses the membrane electric field. Serine substitution of the S4 R3 (R531) residue results in *ω* currents for mutant hERG channels at hyperpolarized membrane potentials. The gating pore of this mutant hERG channel conducts cations as large as guanidinium (Kudaibergenova et al, 2022). These findings suggest that the S4 R3 residue is near the HCS of hERG channels at hyperpolarized membrane potentials.

Without domain swap, packing of the voltage sensor domain against the pore domain of the same subunit (Wang and MacKinnon, 2017) may lead to noncanonical electrochemical coupling for hERG channel activation. Indeed, splitting hERG at the end of the S4–S5 linker has little effect on channel activation, while these split hERG channels display accelerated deactivation (de la Peña et al, 2018b). Splitting hERG within the long S2-S3 linker also has little effect on S4 translocation across the membrane for channel activation (de la Peña et al, 2018a). Closure of hERG channels may involve interactions of the pore domain with the cytoplasmic end of S4 and the initial section of the S4–S5 linker (de la Peña et al, 2018b), as well as the interaction between the S4 K538 residue and the S1 D411 residue (Dou et al, 2017). These findings support the notion that electrochemical coupling for hERG channel gating depends on physical contact between the S4 voltage sensor and the pore domain rather than the connection of S4 with the pore domain via the S4–S5 linker.

## 9 The Eag1 (Kv10.1) voltage-gated potassium channel

The Eag1 (Ether-à-go-go 1, Kv10.1) channel activates slowly and shows no inactivation. It is normally expressed in multiple brain regions and frequently detected in tumor biopsies (Luis et al, 2022). The Eag1 channel with a short S4–S5 linker displays extensive contact between the voltage sensor domain and the pore domain. For example, S1 of the voltage sensor domain packs against S5 of the pore domain of the same subunit (Whicher and MacKinnon, 2016). Without domain swap between the voltage sensor domain and the pore domain, the Eag1 channel has the N-terminal Per-ARNT-Sim (PAS) domain of 1 subunit interacting with the C-terminal cyclic nucleotide–binding homology domain of a neighboring subunit, with the N-terminus of the PAS domain directed toward the voltage sensor (Whicher and MacKinnon, 2016). The Eag1 channel with bound calmodulin has a closed pore, with the C-terminus of S4 directed toward the C-linker that follows S6 (Whicher and MacKinnon, 2016).

Cryo-EM analyses of channels in membrane vesicles allow examination of their voltage sensors at different membrane potentials. Studies of liposomes containing Eag1 channels associated with Ca^2+^-bound calmodulin reveal S4 arrangements in the activated state, an intermediate state, as well as a presumed resting state (Mandala and MacKinnon, 2022) (Fig. 2). For voltage sensors in the activated (depolarized) state of Eag1 channels that are either suspended in detergent or reconstituted in liposomes, the S4 basic residues K1 (K327), R2 (R330), and R3 (R333) interact with the S2 D258 and D254 residues above the HCS. The S4 R4 (R336) residue resides in the HCS, which contains the S2 F261 and D264 residues and the S3 D299 residue. The S4 R5 (R339) residue is in the proximity of the S3 W295 and D299 residues, and the S4 K6 (K340) residue is near the S1 D221 and W222 residues (Mandala and MacKinnon, 2022).

Eag1 channels in liposomes with a voltage across the membrane may have their voltage sensors in the intermediate state or the resting (hyperpolarized) state, with S4 shifting progressively downwards and forming a more extended interfacial helix near the inner leaflet of the membrane. For the voltage sensor in the intermediate state, the S4 K1 and R2 residues are above the HCS, R3 is in the HCS, while R4 and R5 are below the HCS. For the voltage sensor in the resting (hyperpolarized) state, K1 is above the HCS and R2 is in the HCS, while R3, R4, and R5 are below the HCS (Mandala and MacKinnon, 2022) (Fig. 2). These structural analyses provide evidence for stepwise S4 translocation through the HCS.

A physical connection between the voltage sensor domain and the pore domain via the short S4-S5 linker is not required for voltage gating of Eag1 channels, as in the case of hERG channels. Eag1 with its pore domain split from the voltage sensor domain, still forms voltage-gated potassium channels. The presence of the voltage sensor domains appears to be structurally important for the formation of a functional channel pore (Lörinczi et al, 2015; Luis et al, 2022). Unlike breaks in the S4–S5 linker, breaks within S4 result in constitutively active channels, suggesting that channel closing may involve motions of the C-terminal S4 helix (Luis et al, 2022).

The voltage sensor domain interacts with not only the pore domain but also the cytoplasmic domain of Eag1 channels. Interaction between D342 at the C-terminal end of S4 and the cytoplasmic PAS loop N-terminus appears to stabilize the voltage sensor in the activated (depolarized) state. This interaction further promotes channel opening by destabilizing interactions involving the cytoplasmic PAS loop C-terminus, Y213 of the voltage sensor domain, and Y639 of the cytoplasmic cyclic nucleotide–binding homology domain. S4 movements driven by hyperpolarization disrupt the interaction between the S4 D342 residue and the cytoplasmic PAS loop N-terminus. Consequently, the cytoplasmic PAS loop C-terminus may interact with Y213 and Y639 to promote channel closing, which can be stabilized by calmodulin binding to the cytoplasmic domains (Whicher and MacKinnon, 2019).

## 10 The HCN cation channels

Hyperpolarization and cAMP binding activates the HCN channel for the permeation of Na^+^ and K^+^ ions, leading to slow depolarization of cardiac pacemaker cells for action potential firing (Porro et al, 2020). Of the 4 HCN family members in mammals, HCN1 channels open at the most depolarized membrane potential and display the smallest cAMP-induced shift of voltage dependence, while HCN4 channels open at the most hyperpolarized membrane potential and display the largest cAMP-induced shift of voltage dependence (Porro et al, 2020).

The HCN1 channel displays no domain swap so that the voltage sensor domain is adjacent to the pore domain of the same subunit. Binding of cAMP results in a rotation of the cytoplasmic domains (Lee and MacKinnon, 2017). Compared with HCN1 channels in the depolarized state, S4 shifts downward by 2 helical turns in the hyperpolarized state of HCN1 channels, which can be stabilized via metal affinity bridging. The 3_10_ helical segment of S4 remains at the center of the membrane for HCN1 channels in a depolarized or hyperpolarized state, albeit with different amino acid compositions (Lee and MacKinnon, 2019) (Fig. 2).

With the concertina effect of S4 rearrangements from an *α* helix to a 3_10_ helix during S4 translocation through the HCS in response to hyperpolarization, the S4 basic residues turn away from the lipid headgroups as they move downward into the HCS. In the depolarized state, the HCS is below K1 (K261), S2 (S264), R3 (R267), and R4 (R270) but above R5 (R273) and R6 (R276) on S4. In the hyperpolarized state, the HCS is below K1 and S2 but above R3 and R4, which are above a break of the S4 helix stabilized by hydrogen bond formation between the S4 S272 residue and the carbonyl oxygen of the S4 L269 residue (Lee and MacKinnon, 2019) (Fig. 2).

Breaking the S4 helix during its downward movement may trigger a series of interactions to cause the HCN1 channel pore to open in the hyperpolarized state. The lower S4 helical segment that is below the break lies horizontally so that the S4 R5 and R6 residues face the intracellular solution while several S4 hydrophobic residues face the lipid bilayer (Lee and MacKinnon, 2019). A domino effect, starting with pulling W281 at the end of S4 out of a hydrophobic cavity formed by S1 and the N-terminal HCN domain, may allow the lower S4 helical segment to rotate and establish contact of W281 with the S5 V296 and N300 residues. This sequence of interactions may further cause S5 to bend away from S6, followed by a rotation of the S6 helix with a glycine hinge, thereby causing expansion of the pore (Elbahnsi et al, 2023).

The depolarized state of HCN4 channels resembles that of the HCN1 channels (Fig. 2), with the voltage sensor packed against the pore domain of the same subunit (Saponaro et al, 2021). The S4–S5 linker of one subunit contacts the C-linker of a neighboring subunit of HCN4 channels, with H407 and D411 on the S4–S5 linker joining H553 and E557 on the C-linker of a neighboring subunit to form a tetrad for coordination of a Mg^2+^ ion. These interactions facilitate cAMP modulation of HCN4 channel activity (Saponaro et al, 2021).

Different HCN family members employ a variety of electrochemical coupling schemes. For HCN1 channels, S4 displacement upon hyperpolarization may disrupt contacts between S4 and S5 and cause tilting of S5 (Lee and MacKinnon, 2019). For HCN4 channels, the removal of lipids in between S4 and S5 that appear to be crucial for the interactions among the S4, S5, and S6 helices is associated with an upward tilt of S5 and rotation of S6 for pore opening (Saponaro et al, 2021).

Remarkably, the HCN-like 1 (HCNL1) channel in zebrafish sperm has a nonconductive pore formed by the pore domains, while its 4 voltage sensor domains mediate proton permeation upon membrane hyperpolarization (Wobig et al, 2020). Arginine substitution of the evolutionarily conserved methionine (M169) residue of HCNL1, at the equivalent position of the highly conserved arginine (R248) residue at the N-terminal end of S4 in HCN1 channels, suppresses the proton current while leaving the gating current detectable (Wobig et al, 2020). Proton permeation through the gating pores allows HCNL1 channels to mediate membrane potential regulation of sperm swimming once zebrafish sperm is released into freshwater with very low ion concentrations (Wobig et al, 2020).

## 11 The BK (Slo1) calcium-activated potassium channel

The BK calcium-activated potassium channel with large conductance (also known as Slo1, for Slowpoke 1) has an additional TM segment S0, preceding the voltage sensor domain that is followed by the pore domain. Its cytoplasmic tail domain contains the RCK1 and RCK2 domains that include Ca^2+^ binding sites and form the cytoplasmic gating ring of the tetrameric BK channel (Tao et al, 2017).

The arrangement of S4 voltage sensors in BK channels resembles that of S4 voltage sensors in voltage-gated ion channels. A 3_10_ helix is formed by the S4 segment of BK channels from *Aplysia californica* (acSlo1) and includes the S4 R2 (R196), R3 (R199), and R4 (R202) residues. The S4 R2 and R3 residues form hydrogen bonds with the S1 D120 residue and the S2 D142 residue above the HCS (Fig. 2). The S4 R4 residue interacts with the S2 F149 residue as well as the S3 D175 residue in the HCS that focuses the membrane electric field (Tao et al, 2017).

There is no domain swap between the voltage sensor domain and the pore domain that are connected by a short S4–S5 linker, with S4 packing tightly against S5 of the same subunit in BK channels (Tao et al, 2017). However, the S6–RCK1 linker mediates a domain swap so that the TM domain of 1 subunit is above the cytoplasmic domain of a neighboring subunit (Tao et al, 2017). Ca^2+^ binding causes the cytoplasmic gating ring to compress against the TM domain, leading to the outward displacement of the S5 helices (Hite et al, 2017). Whereas S6 is bent at P309 in the Ca^2+^-free BK channel, S6 is bent at G302, and the RCK1 N-lobes of the gating ring are expanded in the Ca^2+^-bound BK channel, leading to wider pore opening (Hite et al, 2017).

The interplay between electrochemical coupling and calcium gating involves physical contact among the voltage sensor domain, the pore domain, and the cytoplasmic gating ring. The RCK1 N-lobe that is connected to the pore-lining S6 helix via the C-linker also forms an interface with the voltage sensor and the S4–S5 linker. Conformational changes induced by Ca^2+^ binding to the cytoplasmic domain cause the cytoplasmic end of the voltage sensor as well as the S5 helix to move outwards. In the Ca^2+^-bound BK channel, Mg^2+^ ions bridge the RCK1 N-lobe and the TM domain via the S2-S3 linker. This way, each RCK1 N-lobe physically interacts with 2 neighboring subunits upon Ca^2+^ binding and regulates voltage sensing as well as pore opening (Hite et al, 2017).

Mammalian BK channels structurally resemble invertebrate BK channels from *Aplysia* and *Drosophila* (Hite et al, 2017; Tao et al, 2017; Tao and MacKinnon, 2019; Raisch et al, 2021). For mammalian BK channels, the *β* subunits with 2 TM helices contact the voltage sensors of 2 neighboring subunits (Tao and MacKinnon, 2019). In addition, the *ɣ* subunit with 1 TM helix fits into a cleft formed by S0 as well as the S2 and S3 TM helices of the voltage sensor domain (Redhardt et al, 2024). These auxiliary subunits may further modulate BK channel functions.

Gating current measurements indicate that the gating charge of mouse BK channels, which is about one-fifth of the gating charge of Kv1 voltage-gated potassium channels, is carried by the S4 R3 (R210) and R4 (R213) residues. Histidine substitution of the S4 R3 residue results in hyperpolarization-induced *ω* currents owing to proton permeation, indicating that R3 is near the HCS (Carrasquel-Ursulaez et al, 2022). At hyperpolarized potential (−80 mV), R3 is exposed to the extracellular solution, while R4 is exposed to the intracellular solution (Hu et al, 2003). It appears that a modest S4 movement generates the gating current of the mouse BK channel (Carrasquel-Ursulaez et al, 2022).

## 12 Voltage-gated potassium channels in plants

Multiple types of voltage-gated potassium channels mediate K^+^ transport in plants and volume control of guard cells involved in stomatal opening (Hedrich, 2012). For KAT1 channels that open slowly upon hyperpolarization to regulate stomatal opening, the voltage sensor domain is adjacent to the pore domain of the same subunit without exhibiting domain swap. The S1 TM helix packs between S4 and S5 of the same subunit near the extracellular side of the membrane, while the intracellular ends of the S4 and S5 helices overlay the C-linker of a neighboring subunit (Clark et al, 2020).

The arrangement of S4 voltage sensors in KAT1 channels resembles that of the S4 voltage sensors in voltage-gated ion channels in animals. The S4 R171 and R174 residues are above the HCS, which contains the S2 F102 and D105 residues and the S3 D141 residue. The S4 R177 residue is below the HCS and interacts with the acidic residues D105 and D141 (Clark et al, 2020; Li et al, 2020) (Fig. 2).

Gating current measurements of KAT1 channels reveal gating charge movements between multiple closed states (Latorre et al, 2003). Cysteine accessibility studies show that cysteines at the positions of the S4 R177 and S179 residues are exposed to the extracellular solution at depolarized but not hyperpolarized potential. In contrast, cysteines at the positions of the S4 R165, R174, and R176 residues are accessible to the extracellular solution at both depolarized and hyperpolarized potential (Latorre et al, 2003). These findings support the notion that hyperpolarization drives downward S4 movement to cause KAT1 channel opening.

The AKT1 inwardly rectifying potassium channels mediate K^+^ transport from the rhizosphere into the root hair (Hedrich, 2012). The voltage sensor domain is packed against the pore domain of the same subunit of AKT1 channels without a domain swap. The S4 R164 and R167 residues are above the HCS that contain the S2 F100 residue. The S4 R169 and R170 residues are below the HCS and interact with the S2 D103 residue, the S3 D139 residue, and the S1 E61 residue (Dickinson et al, 2022) (Fig. 2).

The Stelar K^+^ outward rectifying (SKOR) channels allow K^+^ translocation from the root hair into the xylem for transport across the plant (Hedrich, 2012). The SKOR channel also has a short S4–S5 linker, so that the voltage sensor domain packs against the pore domain of the same subunit. The S4 R185, R191, and R194 residues are above the HCS that contains the S2 F125 residue. The S4 R197 residue is below the HCS and interacts with the S2 D128 residue and the S3 D164 residue (Li et al, 2023a) (Fig. 2). Both SKOR channels and AKT1 channels, which contain 4 pore-forming *α* subunits, display a symmetry reduction to 2-fold symmetry (Dickinson et al, 2022; Li et al, 2023a).

## 13 The 2-pore channel family of ion channels in intracellular organelles

Two-pore channel (TPC) channels in intracellular organelles of plant and animal cells serve a variety of functions. Plant TPC1 channels reside in vacuoles to generate the slow vacuolar current. Mammalian TPC1 and TPC2 channels in endolysosomes control ion homeostasis. TPC channels are formed by 2 *α* subunits that each contain 2 6-TM domains, with S1–S4 (the voltage sensor domain) and S5–S6 (the pore domain) labeled as IS1–S6 and IIS1–S6 in these 2 6-TM domains (Gan and Jiang, 2022; She et al, 2023).

The plant TPC1 channel from *Arabidopsis thaliana*, AtTPC1, is a nonselective cation channel that permeates Ca^2+^ ions. These channels exhibit domain swap so that the voltage sensor of one 6-TM domain interacts with the pore domain of a neighboring 6-TM domain (Guo et al, 2016; Kintzer and Stroud, 2016). Voltage gating depends on R3–R5 of IIS4, which contains 4 basic residues (R1 [R531], R3 [R537], R4 [R540], and R5 [R543]), but not on IS4, which contains 2 arginine residues and forms an *α*-helix (Guo et al, 2016). The AtTPC1 channel activity is also regulated by cytosolic Ca^2+^. The cytoplasmic EF-hand domain of AtTPC1 includes an extension of IS6 and lies just below IS1–S4. This domain undergoes a conformational change upon Ca^2+^ binding, causing movements of the pore-lining IS6 helix (Guo et al, 2016).

AtTPC1 channels are inhibited by luminal Ca^2+^, which binds to a site formed by the IIS1 D454 residue and the IIS4 E528 residue as well as the IS5 D240 residue of the neighboring subunit. Luminal Ca^2+^ binding tethers IIS4 to the static IIS1 and the pore domain of the neighboring subunit, thereby preventing voltage sensor movement and hampering voltage gating (Guo et al, 2016). With the luminal Ca^2+^ binding site occupied to stabilize the resting (hyperpolarized) state, the IIS4 R3 residue is at the HCS that contains the IIS2 Y475 and E478 residues and the IIS3 D500 residue. The S4 R4 and R5 residues are below the HCS in this AtTPC1 channel at the resting (hyperpolarized) state with Ca^2+^-free EF-2 domains (Guo et al, 2016) (Fig. 2).

The voltage sensor is at the activated (depolarized) conformation in the AtTPC1 channel that bears mutations to eliminate the luminal Ca^2+^-binding site but includes Ca^2+^-bound EF-1 and EF-2 domains as well as the Ca^2+^-bound pocket formed by the side chains of D39 and D43 and the main chain carbonyls of D39 and L45 (Ye et al, 2021). The IIS4 R5 (R543) residue is at the HCS. The S4 R3 (R537) and R4 (R540) residues are above the HCS and interact with the IIS1 E450 residue, the IIS2 D468 residue, and the IIS3 E511 residue (Ye et al, 2021) (Fig. 2).

Electrochemical coupling of AtTPC1 channels involves multiple conformation changes triggered by the IIS4 translocation. The IIS4 segment forms a long 3_10_ helix, while its C-terminal segment is bent to run parallel with the membrane and connect to the IIS4–S5 linker that forms extensive contact with IIS6 (Guo et al, 2016). The 3_10_ helix that includes the IIS4 residues R540 to M546 in the activated (depolarized) state is 1 turn shorter than the 3_10_ helix of IIS4 (R537 to M546) in the resting (hyperpolarized) state of AtTPC1 channels (Ye et al, 2021) (Fig. 2). The upward movement of IIS4 driven by depolarization is accompanied with tilting of IIS3, movement of the N-terminal end of IIS4 toward the IS5 helix of the neighboring subunit, rotation of IIS4 around the central pore, and outward rotation of the IIS4–S5 linker, which may cause the pair of IIS6 helices to dilate for channel opening (Ye et al, 2021).

Mammalian TPC1 channels preferably permeate Na^+^ ions and reside in endolysosomes (Gan and Jiang, 2022; She et al, 2023). These channels are activated by depolarization and PI(3,5)P_2_ binding to the first 6-TM domain (She et al, 2018). Like AtTPC1, voltage gating depends on the voltage sensor in the second 6-TM domain, with IIS4 adopting a 3_10_ helical conformation (Fig. 2). There are 2 IIS4 basic residues responsible for voltage gating, R3 (R540) and R5 (R546) of the mammalian TPC1 channel (She et al, 2018).

In the depolarized state of the mammalian TPC1 channel, the IIS4 R3 residue is above the HCS. The IIS4 R5 residue is at the HCS that contains the IIS2 Y487 and E490 residues and the IIS3 D512 residue (Fig. 2). There is an open space between the IIS4–S5 linker and IIS6 to accommodate the PI(3,5)P_2_-induced conformational change of S6 helices for pore opening (She et al, 2018).

Mammalian TPC2 channels also reside in endolysosomes (Gan and Jiang, 2022; She et al, 2023). These channels are activated by PI(3,5)P_2_ but not by membrane depolarization, even though there is a 3_10_ helical segment of IIS4. Of the 2 IIS4 basic residues (R4 (R554) and R5 (R557)), R4 is at the HCS (She et al, 2019). Replacing the IIS4 I551 residue with arginine results in a mutant TPC2 channel with voltage gating (She et al, 2019). Conversely, the substitution of the IIS4 R3 (R540) residue of mammalian TPC1 channels with glutamine or isoleucine transforms the mutant TPC1 channel into a voltage-independent TPC2-like channel (She et al, 2023).

TPC3 channels are in the lysosomes of some mammals but not humans (Gan and Jiang, 2022; She et al, 2023). Voltage gating of TPC3 channels depends on the voltage sensor of the second 6-TM domain (Dickinson et al, 2020). The zebrafish DrTPC3 channel from *Danio rerio* is activated at extreme membrane depolarization. Of the 3 IIS4 basic residues, R3 is above the HCS that contains the IIS2 Y455 residue, while R4 and R5 are below the HCS, with the IIS4 R4 residue forming a cation-*π* interaction with Y455 at the HCS. This IIS4 placement likely corresponds to the resting (hyperpolarized) state of the voltage sensor (Dickinson et al, 2020) (Fig. 2).

## 14 Key differences in electrochemical coupling via voltage sensors of different ion channels

For voltage-gated sodium channels and calcium channels that are formed by a large *α* subunit with 4 homologous 6-TM domains and display domain swap, the 4 voltage sensors may play different roles in channel gating. For example, S4 in *D*IV is involved in fast inactivation rather than activation of voltage-gated sodium channels (Goldschen-Ohm et al, 2013; Jiang et al, 2021b). For TPC1 channels that also exhibit domain swap and are formed by 2 *α* subunits each with 2 homologous 6-TM domains, S4 in the second 6-TM domain serves as the voltage sensor (Guo et al, 2016; Kintzer and Stroud, 2016; She et al, 2018). As to heterotetrameric ion channels formed by 4 homologous rather than identical *α* subunits each with 1 6-TM domain, it remains an intriguing open question whether the 4 voltage sensors may differ in their functional roles.

Voltage-gated potassium channels in the Kv1–Kv9 families display domain swap and have S4–S5 linkers longer than those of potassium channels in the Kv10–Kv12 families (Whicher and MacKinnon, 2016, 2019). These potassium channels with domain swap may employ the canonical electrochemical coupling via interactions between the S4–S5 linker and the cytoplasmic end of the pore-lining S6 helix for voltage sensor movements to control pore opening (Blunck and Batulan, 2012). There is no domain swap between the voltage sensor domain and pore domain of voltage-gated potassium channels in the Kv10–Kv12 families, the BK calcium-activated potassium channel, the HCN family of HCN cation channels, and plant voltage-gated potassium channels that are activated by hyperpolarization. The electrochemical coupling in these channels without domain swap may rely primarily on the physical contact between the voltage sensor domain and the pore domain rather than their connection via the S4–S5 linker (Lörinczi et al, 2015; de la Peña et al, 2018b). These channels may be further modulated by calcium ions and signaling molecules such as cyclic nucleotides (Hite et al, 2017; Porro et al, 2020; Saponaro et al, 2021). Whereas membrane potential changes dictate the direction of S4 voltage sensor movements, variations of electrochemical coupling allow some channels to be activated by hyperpolarization rather than depolarization. For example, breaking the lower part of the S4 helix during its downward movement may trigger a series of interactions to open the HCN1 channel pore upon hyperpolarization (Lee and MacKinnon, 2019; Elbahnsi et al, 2023).

## 15 The Hv1 voltage-gated proton channel

Voltage-gated proton (Hv) channels emerged in protists during evolution; these channels are not found in eubacteria or archaea. Hv channels share 28%–36% amino acid identity (∼50%–60% similarity) with eukaryotic voltage-gated sodium channels and calcium channels, indicating that Hv channels may have evolved from a split of the voltage sensor domain from an early eukaryotic sodium channel or calcium channel (DeCoursey, 2018; Chaves et al, 2023). Hv channels form dimers in mammals, insects, ascidians, sea urchins, and reef-building corals (Chaves et al, 2023). Many eukaryotes have 1 gene for Hv channels, while several marine species that are capable of biomineralization have multiple genes for Hv channels. Notably, some eukaryotes, including all modern insects (Diptera) and most of the nematodes, lack Hv channels (Chaves et al, 2023).

Gating current measurements indicate that a conformational rearrangement that follows voltage sensor activation is required to open the Hv1 channel (De La Rosa and Ramsey, 2018). All 3 S4 basic residues contribute to the gating charge (DeCoursey, 2018).

Mammalian Hv1 voltage-gated proton channels open slowly. The acidic residue D112 in the middle of S1 is crucial for proton selectivity (Musset et al, 2011). In the open channel, the S1 D112 residue may interact with the S4 R3 (R211) residue (Berger and Isacoff, 2011). In the closed channel, D112 switches to interact with the S4 R1 (R205) residue instead (Takeshita et al, 2014). Moreover, the S4 R1 (R205) and R2 (R208) residues of the open channel are accessible to the extracellular solution, while the S4 R2 residue of the closed channel is accessible to the intracellular solution. The S4 R3 residue is only accessible to the intracellular solution in open or closed channels (DeCoursey, 2018). In support of the notion that the S4 R1 residue may be in the vicinity of the HCS in the closed channel, histidine substitution of the S4 R1 residue results in proton leakage in closed Hv1 channels (Randolph et al, 2016). In support of the notion that the S4 R3 residue may be in the vicinity of the HCS in the open channel, R3 mutations enable guanidinium ions to go through the Hv1 channel (Berger and Isacoff, 2011). These studies indicate that a downward S4 movement closes the Hv1 channel.

A closed conformation of Hv1 channels has been determined via crystallography studies of the mouse Hv1 channel with part of its S2 and S3 segments replaced with the corresponding region of the *Ciona intestinalis* VSP (Ci-VSP) and its C-terminal coiled-coil region replaced with a leucine-zipper motif of the GCN4 yeast transcription factor (Takeshita et al, 2014). Zn^2+^ binding at a site coordinated by 2 S2 acidic residues as well as an S2 histidine residue and a histidine residue in the S3–S4 linker inhibits Hv1 channel opening, likely by preventing the upward movement of S4 (Takeshita et al, 2014). In this resting (hyperpolarized) state, the S4 R2 and R3 residues are below the HCS. The S4 segment that includes R1 and R2 forms a 3_10_ helix (Takeshita et al, 2014) (Fig. 2). Notably, the conserved S2 phenylalanine (F150 in human Hv1) in the HCS plays a key role in interacting with the guanidinium derivative 2GBI, a modulator that can only access the open Hv1 channel from the intracellular side of the membrane to block the channel and to slow channel closing (Hong et al, 2013).

The human TM protein with unknown function 266 (TMEM266) resembles Hv1 in having a voltage sensor domain composed of S1–S4 with an extension of S4 to include a coiled-coil segment for dimerization, as well as a Zn^2+^ binding site on the extracellular side of the voltage sensor domain (Papp et al, 2019). The hTMEM266 protein is localized to the dendrites of cerebellar granule cells, at the postsynaptic site that is in synaptic contact with glutamatergic mossy fiber terminals (Kim et al, 2014). TMEM266 is highly conserved in vertebrates and, like Hv1, is more closely related to voltage-gated sodium channels than other voltage-gated ion channels. However, the hTMEM266 protein does not appear to form an ion channel, and its function remains unknown (Kim et al, 2014).

## 16 The sperm-specific Na^+^/H^+^ exchanger SLC9C1 with a voltage sensor

The sperm-specific solute carrier SLC9C1 is a Na^+^/H^+^ exchanger essential for male fertility. It has a unique domain composition, including a transporter domain with 13 TM helices, a voltage sensor domain of 4 TM segments (S1–S4), and a cytoplasmic domain that contains a cyclic nucleotide–binding domain (CNBD) and 9 intracellular helices for interactions between these 3 functional domains. SLC9C1 is activated by hyperpolarization and modulated by cyclic nucleotide (Windler et al, 2018; Kalienkova et al, 2023; Yeo et al, 2023).

Gating current measurements reveal that the S4 R2 (R803) residue contributes to the gating charge. In addition, cAMP binding alters the voltage sensor equilibrium and shifts the voltage dependence of the gating current by 20 mV, thereby modulating the Na^+^/H^+^ exchanger activity (Windler et al, 2018). Cryo-EM analyses of the sea urchin SLC9C1 reveal that it forms a dimer. The voltage sensor domains are at the periphery, separated from the transporter domain by ∼2 nm, while the C-terminal part of the long S4 helix is embedded within the network of intracellular helices (Kalienkova et al, 2023; Yeo et al, 2023).

The arrangement of the S4 voltage sensor in SLC9C1 resembles that of voltage sensors on voltage-gated ion channels. The S4 K1 (K800), R2 (R803), R3 (R806), and R4 (R809) residues are above the HCS, which contains the S2 Y743 and E746 residues and the S3 D767 residue. The S4 R5 (R812) and K6 (K815) residues are below the HCS, with R5 forming salt bridges with the S2 E746 residue and the S3 D767 residue in the HCS (Fig. 2). Above the HCS, the S4 R2 residue forms a salt bridge with the S1 E698 residue. The S4 R3 residue forms a salt bridge with E698 while hydrogen bonding with the S2 N736 residue, and the S4 R4 residue forms a hydrogen bond with the S1 N691 residue (Kalienkova et al, 2023; Yeo et al, 2023).

SLC9C1 is structurally closely related to voltage-gated sodium channels. With its S4 segment including residues from R2 (R803) to K6 (K815) in a 3_10_ helical conformation, the SLC9C1 voltage sensor domain resembles the voltage sensor domain in *D*IV of the human Na_V_1.7 voltage-gated sodium channel in the depolarized state (Yeo et al, 2023). The binding of cAMP to the CNBD domain, which enables SLC9C1 activation via less hyperpolarization, is associated with a rotation of S1–S3, similar to the rotation of S1–S3 that accompanies the vertical displacement and unwinding of the S4 helix in *D*I of the Nav1.7 channel bearing mutations that cause right shifts of the voltage dependence of activation (Shen et al, 2019; Huang et al, 2022; Yeo et al, 2023). Thus, cAMP binding primes SLC9C1 activation by detaching CNBD interactions with the cytoplasmic end of S4 (Yeo et al, 2023).

## 17 VSPs

VSPs respond to membrane potential changes and dephosphorylate PI signaling lipids (Murata et al, 2005; Iwasaki et al, 2008; Halaszovich et al, 2009; Matsuda et al, 2011; Kurokawa et al, 2012; Sakata and Okamura, 2014). The voltage sensor of Ci-VSP from *Ciona intestinalis* moves in 2 stages with progressive depolarization, each causing an increase in the phosphatase activity (Sakata and Okamura, 2014). The substrate preference of Ci-VSP shifts from phosphatidylinositol (3,4,5)-trisphosphate (PIP_3_) at low-voltage depolarization to PIP_2_ at high-voltage depolarization (Grimm and Isacoff, 2016).

Voltage sensor mutagenesis studies support the notion that as S4 moves upwards, it first reaches the intermediate state at low-voltage depolarization and then to the fully activated state at high-voltage depolarization, thereby rendering Ci-VSP with different substrate preferences. Tryptophan substitution of the S2 F161 residue at the HCS combined with lysine substitution of the S4 R4 (R232) residue stabilizes the intermediate conformation of the voltage sensor that is associated with the PIP_3_ substrate preference while strongly suppressing the fully activated state that is associated with a preference for PIP_2_ as the Ci-VSP substrate. In contrast, alanine substitution of the W182 residue near the cytoplasmic end of S4 causes a left shift of both the voltage dependence of Ci-VSP activation to the PIP_2_-preferring intermediate state and the voltage dependence of Ci-VSP activation to the PIP_3_-preferring fully activated state (Grimm and Isacoff, 2016).

Structural analyses have been carried out for the voltage sensor of Ci-VSP with glutamate substitution of the R217 residue at the N-terminal end of S4, which shifts the voltage dependence of the gating current (Villalba-Galea et al, 2013; Li et al, 2014). At 0 mV, the R217E mutant Ci-VSP is in the intermediate state with a preference for PIP_3_ as the substrate (Grimm and Isacoff, 2016). Crystallography studies of the Ci-VSP voltage sensor domain bearing the R217E mutation reveal that the S4 R1 (R223) and R2 (R226) residues are above the HCS, which contains the S1 I126 residue, the S2 F161 residue, and the S3 I190 residue. The S4 R3 (R229) residue is at the level of the HCS, while R4 (R232) is below the HCS (Fig. 2). These S4 basic residues may interact with the S3 E183 and D186 residues below the HCS and the S1 D129 residue above the HCS (Li et al, 2014). The ion pairs likely switch partners while the S4 basic residues move through the HCS (Li et al, 2014; Shen et al, 2022; Guo et al, 2024).

The VSP voltage sensors are closely related to the Hv1 voltage-gated proton channels. However, Ci-VSP does not conduct proton currents (Musset et al, 2011). Histidine or cystine substitution of the S4 R1 (R223) residue of Ci-VSP leads to proton permeation at hyperpolarized potentials (Villalba-Galea et al, 2013; Shen et al, 2022). Upon voltage sensor activation, Ci-VSP with histidine substitution of the S4 R2 (R226) or R3 (R229) residue mediates proton permeation, while histidine in the position of the S4 R4 (R232) residue becomes accessible to the extracellular solution (Villalba-Galea et al, 2013). Moreover, neutralization of the conserved S1 D129 residue just above the HCS causes a right shift of the voltage sensitivity for voltage sensor activation and leads to anion-selective *ω* currents (Shen et al, 2024). These findings support the notion that the electric field is focused on a narrow hydrophobic region for the translocation of S4 gating charges of Ci-VSP.

VSP family members in different species display different expression patterns for a variety of functions. The Ci-VSP from the sea squirt sperm, as well as VSPs from zebrafish and amphibians, are subject to regulation by the voltage across the cell membrane (Murata et al, 2005; Hossain et al, 2008; Ratzan et al, 2011). The mammalian orthologs TM phosphatase with Tensin homology (TPTE) and TPTE2 are localized to the Golgi complex of spermatogenic cells, with phosphatase activity intact for TPTE2 but not TPTE (Leslie et al, 2007). Mammalian orthologs of VSPs typically have histidine at the end of S4, a position that is occupied by arginine for VSP/TPTE proteins from fish, birds, reptiles, amphibians, and the platypus. The voltage sensors of these mammalian VSP orthologs may conceivably conduct proton currents at strongly positive membrane potentials of the Golgi complex or the Golgi-derived acrosome released from sperm (Sutton et al, 2012).

## 18 Principles for engineering a voltage sensor

Voltage sensors composed of 4 TM segments (S1–S4) have been found in a wide variety of voltage-gated ion channels as well as the sperm-specific Na^+^/H^+^ exchanger and VSPs. To better understand the principles that enable voltage sensors to accomplish the remarkable feat of responding to membrane potential changes by moving charged residues across the membrane electric field, one may examine features that have been preserved during evolution.

Analyses of thousands of voltage sensor sequences reveal strong evolutionary coupling at the S1–S2 interface and the S2–S3 interface. This evolutionarily coupled network of residues appears to form a hydrophobic net to constrain the geometry of the HCS formed by S1–S3 (Palovcak et al, 2014; Toombes and Swartz, 2014). This finding supports the notion that the stable scaffold at the HCS focuses the membrane electric field that drives S4 translocation. The HCS typically includes an aromatic residue and 2 acidic residues that could interact with S4 gating charges as these positively charged residues move through the gating pore formed by the HCS (Long et al, 2005, 2007; Chen et al, 2010; Tao et al, 2017; She et al, 2018; Dickinson et al, 2020; Taylor et al, 2020; Li et al, 2021a,b; Botte et al, 2022; Chi et al, 2022; Mandala and MacKinnon, 2022; Tan et al, 2022; Y. Wu, preprint, DOI: https://doi.org/10.1101/2023.06.02.543446).

Sequence analyses identify another network of coevolving bulky residues at the base of the S1 and S2 helices, which could form a cage to stabilize acidic residues below the HCS (Palovcak et al, 2014). In addition, there are 2 coevolving pairs of residues at the hyperpolarized state of the voltage sensor. These findings indicate that the same S4 arginine residue may interact with either an S1 polar residue or an S2 acidic residue (Palovcak et al, 2014; Toombes and Swartz, 2014). There appears to be evolutionary conservation of the voltage sensor features, including a hydrophobic constriction for translocation of S4 gating charges, as well as polar residues for electrostatic interactions that may stabilize these gating charges.

The gating charges predominantly correspond to S4 arginine residues with very high pKa (∼13.8), because of the delocalization of the positive charge within the guanidinium sidechain. Unlike arginine, the positive charge of lysine is focused on the terminal aliphatic amino group, so that its aqueous pKa is ∼10.5 (Armstrong et al, 2016). The preference for arginine may be accounted for by differences in the hydration properties of these 2 positively charged residues. The arginine guanidium group is poorly hydrated above and below the plane. This feature could facilitate arginine translocation through the HCS. In contrast, the spherical hydration structure of the terminal amino group of lysine may be less amenable to movement through a hydrophobic environment (Thanki et al, 1988; Mason et al, 2003).

A comprehensive survey of the protein data bank supports the notion that arginine has favorable features for the voltage sensor function (Armstrong et al, 2016). Arginine may be more suitable for serving the role of a mobile gating charge because of the segregation of the out-of-plane nonpolar surfaces and the capacity for in-plane polar interactions. The guanidinium sidechain of arginine could have in-plane polar interactions with oxygen-hydrogen bond acceptors of amino acids that contain the carboxylate or hydroxyl moiety. This chemical feature allows arginine to slide through a HCS while maintaining in-plane charge solvation (Armstrong et al, 2016).

The same evolutionarily conserved feature applies to voltage sensors of voltage-gated sodium channels, calcium channels, potassium channels, nonselective cation channels, the sperm-specific Na^+^/H^+^ exchanger, and VSPs (Fig. 2). This feature allows S4 arginine to go through the HCS while preventing ion flow through this gating pore. Notably, replacing S4 arginine that has a guanidinium sidechain with amino acids with a smaller sidechain could result in *ω* currents through the gating pore; without S4 arginine in the HCS, guanidinium ions could go through the gating pore owing to their hydration properties (Armstrong et al, 2016). As a counterexample to this feature with evidence of evolutionary conservation in all kingdoms of life (Yu and Catterall, 2004; Palovcak et al, 2014), voltage-gated proton channels rely on the gating pore of the voltage sensor domain for proton permeation—a twist of the voltage sensor characteristics that emerged in eukarya during evolution (DeCoursey, 2018; Wobig et al, 2020; Chaves et al, 2023).

## Conflict of interest

The authors declare no conflicts of interest.
